# Unraveling Resistance Mechanisms to Gαq Pathway Inhibition in Uveal Melanoma: Insights from Signaling-Activation Library Screening

**DOI:** 10.3390/cancers18010074

**Published:** 2025-12-25

**Authors:** Simone Lubrano, Rodolfo Daniel Cervantes-Villagrana, Nadia Arang, Elena Sofia Cardenas-Alcoser, Kuniaki Sato, Gabriela Cuesta-Margolles, Justine S. Paradis, Monica Acosta, J. Silvio Gutkind

**Affiliations:** 1 Moores Cancer Center, University of California San Diego, La Jolla, CA 92093, USArcervantesvillagrana@health.ucsd.edu (R.D.C.-V.); nadia.arang@ucsf.edu (N.A.); ecardenasalcoser@health.ucsd.edu (E.S.C.-A.); gcuestamargolles@health.ucsd.edu (G.C.-M.);; 2 Department of Pharmacology, School of Medicine, University of California San Diego, La Jolla, CA 92093, USA; 3 Quantitative Biosciences Institute (QBI), University of California San Francisco, San Francisco, CA 94158, USA

**Keywords:** uveal melanoma, resistance, MEK, FAK, AKT, mTOR, BCL-XL, YAP/TEAD, hippo pathway

## Abstract

Background/Objectives: Uveal melanoma (UVM), the leading primary intraocular cancer in adults, is driven by *GNAQ*/*GNA11* mutations, encoding the active forms of Gαq proteins. While local treatments like surgery or radiation can control primary tumors, nearly half of patients die from metastasis. Our aim was identifying potential pathways involved in resistance to targeted therapy in UVM. Methods: Here, we screened 100 pathway-activating mutant complementary DNAs by lentiviral overexpression to identify those that enhance the survival of cancer cells in the presence of clinically relevant targeted therapies, using *BAP1* wild-type UVM cells and validated the most significant results in *BAP1*-mutant cells. Results: This revealed JAK/STAT activation, overexpression of anti-apoptotic BCL2/BCL-XL, and dysregulated PI3K/mTOR or Hippo pathways as escape routes under MEK-ERK or FAK inhibition. Bioinformatic analysis of UVM transcriptome in TCGA further showed that high expression of the hallmark PI3K/AKT/mTOR pathway and IL6/JAK/STAT signaling correlates with poor prognosis. A similar correlation was shown by YAP and anti-apoptotic signatures. The analysis of individual representative genes from these signatures revealed that *MTOR*, *BCL2L1* (BCL-XL), and *TEAD4* gene expression are linked to poorer survival, underscoring the potential clinical impact of these adaptive pathways. Proliferation and apoptosis assay demonstrated that aberrant activation of AKT and YAP promotes resistance to FAK and MEK inhibitors. Conclusions: These findings support the adaptability of UVM lesions and suggest rational combination therapies targeting both primary *GNAQ*/*GNA11*-driven oncogenic signals and their compensatory networks as a more effective, personalized treatment approach for advanced UVM.

## Simple Summary

Uveal melanoma is an aggressive ocular malignancy with high mortality rates. Although localized interventions such as surgical resection or radiotherapy effectively control the primary tumor, many patients ultimately develop metastatic disease. Current therapies targeting principal oncogenic drivers often fail to provide durable responses, underscoring the need to elucidate alternative survival pathways that confer resistance. In this study, we systematically evaluated gene function in laboratory models of uveal melanoma, identifying critical contributions from signaling pathways including JAK/STAT, BCL2/BCL-XL, PI3K/mTOR, and Hippo. These results highlight novel molecular mechanisms underpinning therapeutic resistance and support the development of combinatorial treatment strategies. Such approaches may offer improved clinical outcomes for patients with advanced uveal melanoma by effectively targeting both primary oncogenic drivers and resistance mechanisms.

## 1. Introduction

Uveal melanoma (UVM) originates from melanocytes within the uveal tract (choroid, ciliary body, and iris), and it is the most common primary intraocular malignancy in adults [1], with an incidence of approximately 5 cases per million per year [2]. Despite modern surgical, laser, and radiotherapeutic modalities achieving satisfactory local tumor control, nearly half of patients eventually develop metastatic disease, most notably in the liver, where survival typically drops to less than 12 months from the time of diagnosis [3]. Current systemic therapies offer only limited benefit in the metastatic setting [4,5], highlighting an urgent need for strategies to overcome intrinsic and acquired therapy resistance mechanisms, and thereby improve patient treatment outcomes.

In contrast to cutaneous melanoma, which frequently harbors UV-induced mutations in *BRAF* and *NRAS* [6,7], UVM is driven by distinct oncogenic events that reflect a different pathophysiological profile. The majority of UVM cases (over 90%) harbor mutations in *GNAQ* or *GNA11*, which encode Gαq subunits that instigate constitutive signaling through downstream pathways such as MEK-ERK and YAP [8,9,10]. These alterations create a tumor microenvironment and signaling architecture distinct from other melanoma subtypes, emphasizing the need for specialized therapeutic approaches. A particularly notable genetic event involves the inactivation of the tumor suppressor BAP1 in nearly 50% of patients, which strongly correlates with a high-risk, metastatic phenotype [11]. This leads to dysregulated DNA damage responses, driving tumor aggressiveness and disease progression; however, how these changes integrate with or modulate critical survival pathways remains only partially understood. Recent studies have highlighted the centrality of focal adhesion kinase (FAK) [12] in the context of oncogenic *GNAQ*/*GNA11* alterations, which converge on FAK, AKT, and YAP activation concomitant with components of the MEK-ERK axis to initiate proliferative signals, maintain cell viability, and promote resistance to targeted therapies. Nonetheless, the intricate interplay among these nodal pathways and how they collectively establish a dynamic, adaptive signaling circuit capable of evading single-agent therapies remains poorly defined.

Targeted therapies have revolutionized cancer treatment by selectively inhibiting oncogenic drivers. However, a persistent and difficult challenge is the ability of tumor cells to engage compensatory bypass pathways that neutralize or circumvent the inhibitory effects of these agents. Specifically, the prolonged blockade of signaling hubs, such as EGFR, PI3K, or BRAF, has been shown to activate intricate feedback loops that disrupt or rewire signal transduction, ultimately conferring therapeutic resistance and promoting disease progression [13,14,15,16,17]. In response, there is a growing consensus that combinatorial strategies targeting both the principal oncogenic node and its critical compensatory pathways are necessary to achieve sustained clinical benefit [18]. Yet major questions remain unanswered regarding which Gαq–regulated routes underlie resistance to canonical (e.g., MEK inhibitors, MEKi [19,20,21]) and non-canonical (e.g., FAKi [8,12,22]) targeting agents, and which feedback mechanisms should be precisely co-targeted to optimize therapeutic efficacy. Specifically, despite preclinical evidence that MEKi and FAKi may reduce UVM cell proliferation, these interventions often yield cytostatic rather than cytotoxic responses. Over time, the surviving tumor cells acquire or exploit alternative molecular programs, rendering the tumor refractory and enabling tumor progression or relapse after an initial favorable response. In the present study, we used a recently described “signaling toolkit” strategy to identify mechanisms of resistance to MEKi or FAKi, and explored the status of activation of the emerging adaptive resistance pathways by transcriptome analysis of large RNA sequencing datasets from UVM lesions.

## 2. Materials and Methods

### 2.1. Cell Lines, Culture Procedures and Chemicals

HEK293 cells were maintained in DMEM (D6429, Sigma-Aldrich Inc., St. Louis, MO, USA) supplemented with 10% FBS (F2442, Sigma-Aldrich Inc., St. Louis, MO, USA), 1X antibiotic-antimycotic solution (A5955, Sigma-Aldrich Inc., St. Louis, MO, USA), and 1X Plasmocin prophylactic (ant-mpp, InvivoGen, San Diego, CA, USA). Uveal melanoma cell line 92.1 was maintained in RPMI-1640 medium (R8758, Sigma-Aldrich Inc., St. Louis, MO, USA) supplemented with 10% FBS, 1X antibiotic/antimycotic solution, and 1X Plasmocin prophylactic. Uveal melanoma cell line MP46 was cultured in RPMI-1640 medium (R8758, Sigma-Aldrich Inc., St. Louis, MO, USA) supplemented with 20% FBS,1X antibiotic/antimycotic solution, and 1X Plasmocin prophylactic. All the uveal melanoma cell lines were provided by Dr. Andrew Aplin (Thomas Jefferson University, Philadelphia, PA, USA).

Trametinib (GSK1120212) #S2673 and VS-4718 (PND-1186) #S7653 were obtained from SelleckChem (Houston, TX, USA).

### 2.2. Library and Transfections

Lentiviral cDNA library containing 100 distinct both gain-of-function and loss-of-function (dominant-negative) mutants was generously provided by Dr. Wood [23]. All pathway-activating constructs were generated in lentiviral format, and the viruses were produced in HEK293 cells. All 100 distinct lentiviruses were aliquoted and stored at −80 °C. As negative controls, we used the lentiviral HCRed (Addgene, Watertown, MA, USA, 25892) and luciferase (Addgene, Watertown, MA, USA, 25894). To generate cell populations overexpressing each cDNA construct, 92.1 cells were seeded at a density of 500,000 cells per well in a 6-well plate, with one well designated for each lentivirus to be transduced. After 24 h, each well was infected with a single virus from the library at a multiplicity of infection (MOI) of 0.3, ensuring that most infected cells contained only one viral integration. The lentivirus was added directly to the cells in the presence of polybrene (7.5 μg/mL), and the plates were centrifuged at 1200× *g* for 1 h at 37 °C. Twenty-four hours post-infection, the medium was replaced with fresh medium containing puromycin (2 μg/mL) to initiate the selection process. After 48 h of puromycin selection, the cells were split into approximately six equal portions to be frozen at −80 °C and to prepare the viability experiments.

### 2.3. Concentration–Response Curves in Viability Assays

Cells (8000 per well) were plated in 96-well white plates in supplemented medium. For viability screening, cells expressing oncogenic mutants and controls were seeded in parallel. The next day, the cells were treated with increasing inhibitor concentrations. Each inhibitor was tested in seven serial dilutions, with technical triplicates, for 72 h. For trametinib (MEKi), we used 0.003, 0.01, 0.03, 0.1, 0.3, 1, and 10 nM, and for VS-4718 (FAKi), 0.003, 0.01, 0.03, 0.1, 0.3, 1, and 10 μM concentrations in supplemented medium. Cell viability was assessed using the AquaBluer Cell Viability Reagent and measured with a Spark microplate reader (Tecan). Briefly, after 72 h of incubation, the supernatant was aspirated, and supplemental media was added with Aqua Bluer at a 1:100 dilution and incubated for at least 3 h at 37 °C in a 5% CO_2_ atmosphere. GI_50_ values were calculated with GraphPad Prism (v10.4.1, GraphPad Software, Inc., Boston, MA, USA) using a nonlinear regression model (log[inhibitor] vs. response, three-parameter variable slope).

### 2.4. Acridine Orange/Ethidium Bromide Apoptosis Staining

Uveal melanoma cells (92.1 parental, and YAP-5SA and myr-Flag-AKT expressing cells) were seeded in 12-well plates to ~80% confluence and then treated with FAK or MEK inhibitors for 24 h. Cells were washed with 1X PBS and stained with acridine orange/ethidium bromide (50 µg/mL each in PBS). Acridine orange (cat. A3568) and ethidium bromide (cat. 15585011) were obtained from Invitrogen (Carlsbad, CA, USA). Fluorescent images were captured using an inverted microscope (10×) (Echo Revolve, BICO Company, San Diego, CA, USA). Two fields per condition (≥100 cells/field) were imaged in triplicate. Finally, the cells were manually classified as live (green fluorescence) and apoptotic (red and green fluorescence). Acridine orange is permeable in live cells, and ethidium bromide only influxes dying cells.

### 2.5. Proliferation

Cells (10,000 per well) were plated in 96-well clear plates in supplemented medium; parental 92.1 cells and myr-AKT or YAP-5SA expressing cells were seeded in parallel. The next day, the cells were treated in 200 μL supplemented medium with vehicle, FAK inhibitor (0.3 μM, VS-4718), or MEK inhibitor (3 nM, trametinib). Cell confluency was measured using the Cellcyte 3 microscope (Echo, BICO, San Diego, CA, USA) in live cells in real-time during 72 hrs. The analysis of confluency was performed using the Cellcyte 3 software, and the normalized data were plotted.

### 2.6. Western Blot

Cells were seeded in 6-well plates at a density of 300,000 cells (parental cells and myr-AKT or YAP-5SA cells). When they reach an appropriate confluency, cells were serum starved and treated with the FAK inhibitor (1 μM, VS-4718), MEK inhibitor (10 nM, trametinib) for 24 h. For cell lysates, cells were washed with cold 1X PBS and then lysed using Pierce^TM^ RIPA buffer (Thermo Scientific, Waltham, MA, USA, catalog #89900) supplemented with HaltTM Protease and Phosphatase Inhibitor Cocktail (ThermoFisher Scientific, Waltham, MA, USA, 78440) and 1mM sodium orthovanadate (New England Biolabs, Ipswich, MA, USA, P0758S). The lysates were centrifuged at maximum speed at 4 °C, and protein concentrations were determined using DC Protein Assay (BioRad Laboratories, Hercules, CA, USA, catalog #5000111). Finally, lysates were mixed with 4× Laemmli Sample Buffer (BioRad Laboratories, Hercules, CA, USA, 1610747) containing β-mercaptoethanol and boiled for 5 min at 98 °C. The protein samples were resolved by SDS-PAGE gels at 20–30 mAmps and transferred to PVDF membranes (Immobilon-P membranes, Millipore, St. Louis, MO, USA, catalog no. IPV00010) at 25 V overnight. The membranes were blocked with 5% (*w*/*v*) skim milk in 1X TBS-T for 1 hr, and after washing with TBS-T, incubated with the corresponding primary antibodies from Cell Signaling Technologies. Antibodies were used at a 1:1000 dilution: cl-PARP (#5625), ERK1/2 (#9102), pT202/Y204-ERK1/2 (#9101), AKT (#9272), YAP (#14074), and GAPDH (#5174). HRP-conjugated goat anti-rabbit and anti-mouse IgG (Southern Biotech, Birmingham, AL, USA) were applied at a 1:10,000 dilution. Chemiluminescence was detected using Immobilon Western Chemiluminescent HRP substrate (Millipore, St. Louis, MO, USA) and with the ChemiDoc imaging system (Bio-Rad, Hercules, CA, USA). All representative Western blots were conducted in at least 3 independent experiments.

### 2.7. Caspase Activity

We performed CaspaseGlo3/7 assays to measure caspase activity. 92.1 cells were plated at a density of 10,000 cells/well in 96-well white plates one day before the treatments. After 24 h incubation in supplemented 10% FBS medium, the cells were starved and treated with FAK inhibitor (1 μM, VS-4718), MEK inhibitor (10 nM, trametinib), or vehicle control in 100 μL per well. Apoptosis was evaluated 24 h after treatment using the Caspase-Glo 3/7 Assay System (Promega G8090) according to the manufacturer’s guidelines. Briefly, reagents were added to each well with a final dilution of 1:8, and the plates were maintained in darkness at room temperature for 1 hr. Finally, we read the fluorescence in the Spark microplate reader (Tecan, San Jose, CA, USA).

### 2.8. Wound Closure

Cells were seeded on 0.02% gelatin (pre-coated for 1 h) in 12-well plates. After 24 h, the confluent cell monolayers were wounded in the middle with a pipette tip, the cells were washed with 1X PBS to remove the debris on the wound and stimulated with serum-free or supplemental medium (10% FBS). Inhibitors for FAK (1 μM, VS-4718), or MEK (10 nM, trametinib) were incubated for 2 h before wounding and during cell migration. After 18 h, cells were fixed with 4% PFA, stained with crystal violet, washed with PBS, and photographed.

### 2.9. Survival Analysis in TCGA Uveal Melanoma (UVM) Data

RNA-seq and clinical data for the TCGA-UVM dataset were downloaded from the National Cancer Institute Genomic Data Commons using the TCGAbiolinks R package, Release 3.22. Gene signature scores for each patient sample were calculated using the YAP target genes described previously [24] or curated gene sets in MSigDB (https://www.gsea-msigdb.org/gsea/msigdb, accessed 12 December 2025) by single-sample gene set enrichment analysis (ssGSEA) implemented in GenePattern (www.genepattern.org, accessed 12 December 2025). For survival analysis, we first modeled the pathway activity as a continuous variable in Cox proportional hazards regression, utilizing ssGSEA scores generated above. For visualization (Kaplan–Meier plots), patients were divided into “high” and “low” groups, using maximally selected rank statistics as implemented in the survminer R package. This method identifies the optimal cut-off point for a continuous variable, while formally correcting for the multiple testing inherent in scanning all possible thresholds. Unlike naïve repeated log-rank testing, this approach provides adjusted *p*-values and controls type I error. For survival analysis in (see below), the same approach was applied to determine the cutoff for “high” or “low” patient groups utilizing individual gene expression values (Transcripts Per Million; TPM) from TCGA-UVM dataset. All statistical analyses were performed in R version 4.4.1.

### 2.10. Statistical Analysis

All experiments were repeated at least three times. Data are presented as mean ± standard deviation (SD). Multiple comparisons were assessed by one-way or two-way ANOVA following Tukey post hoc, as indicated. Statistical significance in survival curves (Kaplan–Meier plots) was evaluated by the log-rank test. Hazard ratios were computed where relevant. A *p*-value < 0.05 was considered statistically significant. All analyses were performed in GraphPad Prism (Version 10.4.1, GraphPad Software, Boston, MA, USA, www.graphpad.com).

## 3. Results

### 3.1. Unbiased Lentiviral Signaling Pathway Activation Screen in UVM Cells

**Figure 1:**
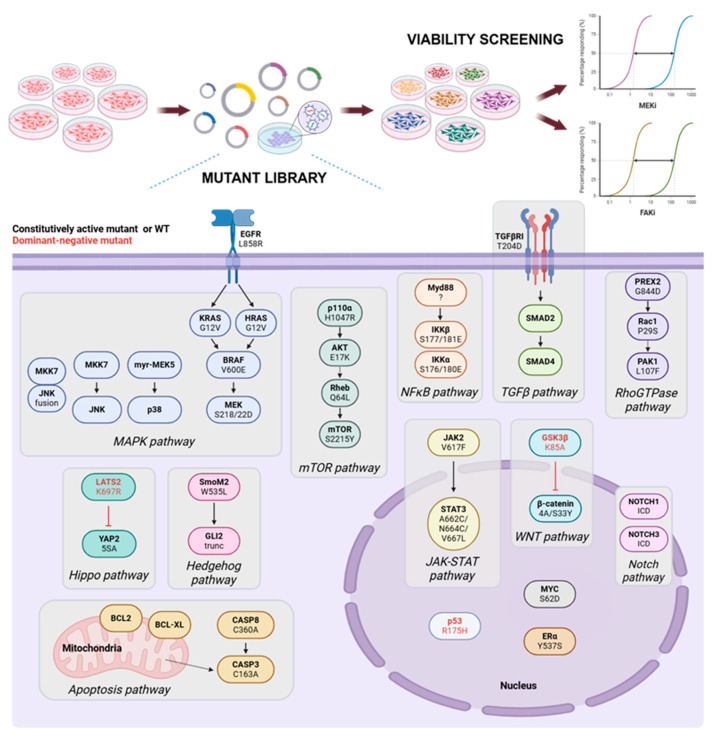
Schematic depicting for mutant library screening to identify resistance mechanisms to MEK or FAK inhibitors. Black, activation (constitutive active mutant or wild type); red, inhibition (dominant negative mutant).

To address the urgent need for a systematic map of resistance-mediating pathways, we first employed an unbiased lentiviral signaling pathway activation screen in 92.1 UVM cells (BAP1 wild-type). This screen was designed to capture a broad spectrum of signaling architecture commonly implicated in oncogenic processes [23]. Specifically, we took advantage of a library of 100 constructs (puromycin-selectable), primarily encoding constitutively active variants of key nodes for signaling pathways known to be pivotal in tumorigenesis (e.g., Ras/MAPK, PI3K/AKT/mTOR, STAT3, Wnt, Notch, TGFβ, Hippo, and oncogenic transcription factors). The library also included a small number of loss-of-function or dominant-negative constructs (e.g., LATS-K697R), whose net effect likewise drives signaling activation. The use of constitutively active mutants, dominant active mutants for pathway repressors, or chimeric fusion active proteins ensured robust and durable signaling output from each node (Figure 1, Table 1).

Each lentivirus was introduced into UVM cells to generate 100 unique lines, each stably overexpressing a single construct. Following puromycin selection, the lines were subjected to full dose–response curves for both MEK (trametinib) and FAK (VS-4718) inhibitors. We derived independent IC_50_ values for each inhibitor and measured cell viability at maximal inhibitory doses (C_max_) (Figure 1). This approach enabled us to capture shifts in dose–response kinetics (i.e., increased IC_50_ values) and any changes in maximal achievable cytostatic or cytotoxic effects.

### 3.2. Emerging Pathways Driving Resistance to MEK and FAK Inhibition

**Figure 2:**
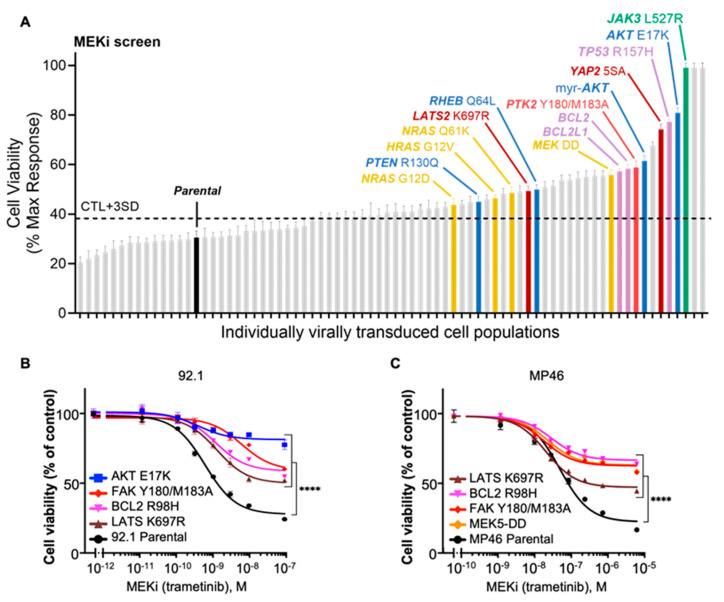
Signaling library toolkit screening reveals candidates as resistance-promoting pathways for MEK inhibition. (**A**) MEKi screen shows cell viability responses of individually virally transduced cell populations in 92.1 cells. Notable resistance mutations include AKT E17K, HRAS G12V, BCL2, and FAK Y180/M183A, which exceed the control threshold. CTL + 3SD: mean viability of control samples plus three times the standard deviation used as a cut-off for resistance. (**B**) Dose–response curves for MEKi (trametinib) in 92.1 cells demonstrate enhanced resistance in populations expressing AKT E17K, BCL2 R98H, LATS K697R, and FAK Y180/M183A compared to parental cells. (**C**) Dose–response curves for MEKi (trametinib) in MP46 cells validate resistance phenotypes driven by LATS K697R, BCL2, FAK Y180/M183A, and MEK5-DD compared to parental MP46 cells. Two-way ANOVA followed by Tukey post hoc, **** *p* < 0.0001.

**Figure 3:**
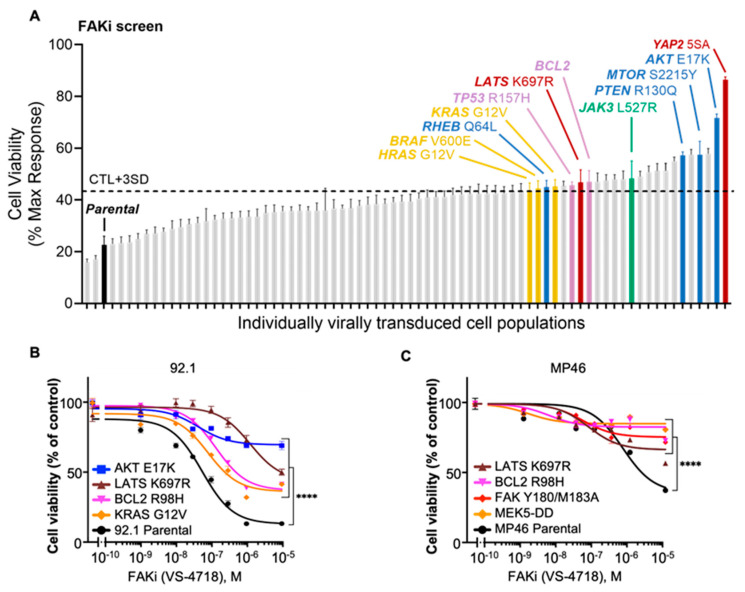
Signaling activation library screening reveals candidates as resistance-promoting pathways for FAK inhibition. (**A**) FAKi screen shows cell viability responses of individually virally transduced cell populations in 92.1 cells. Resistance mutations identified include AKT E17K, mTOR S2215Y, PTEN R130Q, JAK3 L527R, p53 R157H, BCL2, and KRAS G12V, exceeding the control threshold. CTL + 3SD: mean viability of control samples plus three times the standard deviation used as a cutoff for resistance. (**B**) Dose–response curves for FAKi (VS-4718) in 92.1 cells demonstrate resistance phenotypes driven by AKT E17K, BCL2 R98H, LATS K697R, and KRAS G12V compared to parental cells. (**C**) Dose–response curves for FAKi (VS-4718) in MP46 cells confirm resistance driven by LATS K697R, BCL2, FAK Y180/M183A, and MEK5-DD compared to parental MP46 cells. Two-way ANOVA followed by Tukey post hoc, **** *p* < 0.0001.

As anticipated, constructs that augment MAPK signaling emerged prominently in our screens. For example, overexpression of HRAS-G12V or BRAF-V600E, well-established oncoproteins driving ERK hyperactivation, provided robust rescue from MEKi (Figure 2A and Supplementary Table S1). These same constructs also partially conferred protection against FAKi (Figure 3A and Supplementary Table S2). In line with our prior work demonstrating synergy between MEK and FAK dual inhibition in UVM [25], these observations highlight ERK reactivation as a recurrent and potent escape route for *GNAQ*-driven tumor cells, and also reveal that the constitutively active FAK-Y180I/M183A mutant can preserve cellular viability when MEK activity is pharmacologically suppressed (Figure 2A–C).

Beyond the canonical and FAK-centric mechanisms, our unbiased approach identified additional molecular events that can override MEKi or FAKi growth suppression. Overexpression of BCL2 or BCL-XL (also known as BCL2L1) profoundly bypassed the cytostatic effects of either inhibitor. Similarly, the oncogenic gain-of-function mutant p53-R175H decreased sensitivity to both MEKi and FAKi, indicating that the acquisition of p53 alterations (as p53 is rarely mutated in UVM) might be relevant for tailoring therapy and predicting resistance (Figure 3A–C).

We found that PI3K/AKT/mTOR-activating constructs were among the top hits that were rescued from FAKi (Figure 3A–C). For instance, myr-AKT, AKT E17K and mTOR S2215Y, suggest a central and yet underexplored role for PI3K pathway regulation by *GNAQ* via FAK in UVM, which we have recently described based on genome-wide CRISPR-Cas9 screens using FAKi in UVM cells [26]. Interestingly, constitutively active JAK3-L527R and kinase-dead LATS-K697R also emerged as resistance nodes. The LATS-K697R construct, part of the Hippo tumor suppressor pathway, abolishes the LATS kinase function by disrupting the ATP-binding site (lysine-to-arginine mutation), preventing it from phosphorylating and inactivating YAP/TAZ. This loss of LATS activity, in turn, promotes hyperactive YAP/TAZ signaling, offsetting the growth-inhibitory signals triggered by MEK or FAK blockade. Aligned with these results, constitutively active YAP (YAP2-5SA) was also among the top resistance hits.

These findings collectively extend our mechanistic scope, highlighting potential targets that could be explored to optimize therapeutic response. We then validated the most significant hits in MP46 cells (BAP1-mutated), rather than performing a full screen, to confirm that these top resistance mechanisms could similarly emerge despite their distinct genetic background (Figure 2C and Figure 3C).

### 3.3. Gene Set Enrichment Analysis Identifies Key Oncogenic Pathways Correlating with Poor Survival in Uveal Melanoma

Based on the resistance mechanisms identified in our in vitro models under FAK or MEK inhibition, we next sought to determine whether these same pathways are clinically relevant in UVM. To that end, we queried the TCGA UVM cohort (*n* = 80) and performed Gene Set Enrichment Analysis (GSEA) focusing on curated pathway signatures encompassing Hippo/YAP, PI3K/AKT/mTOR, and IL6/JAK/STAT and anti-apoptosis signaling. Specifically, we used publicly available Hallmark and Reactome gene sets from the Molecular Signatures Database (MSigDB) that capture the core components and downstream targets of each pathway [27,28].

**Figure 4:**
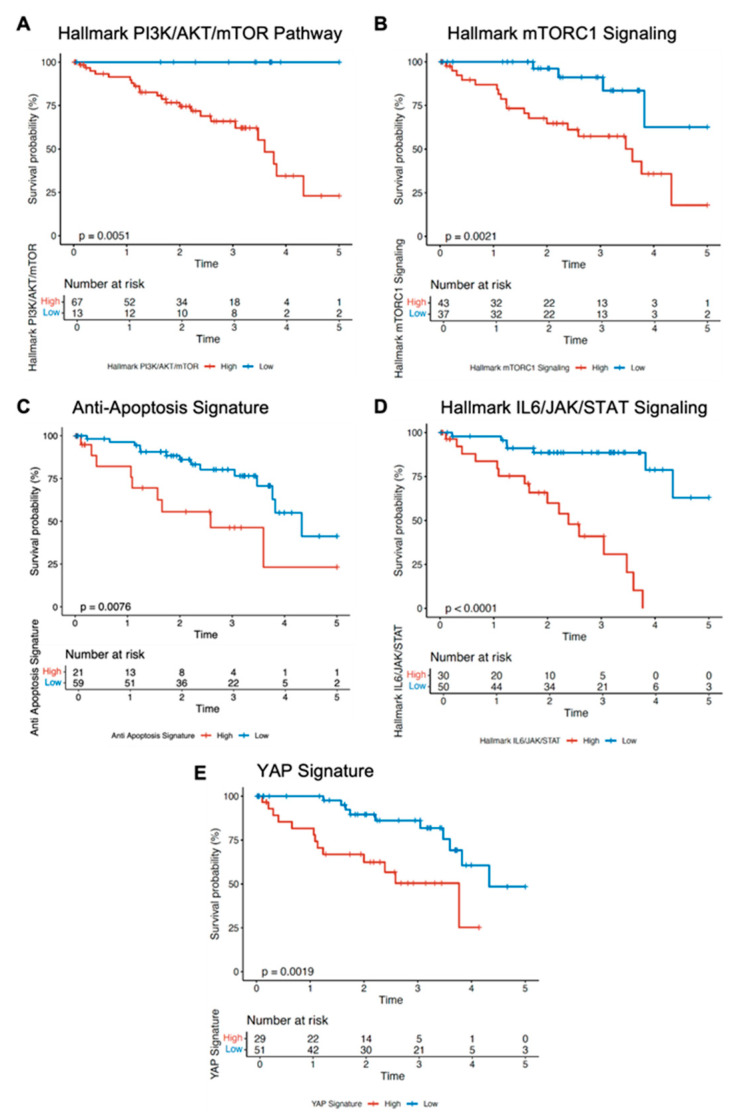
Pathway activity impacts overall survival in uveal melanoma. Gene Set Enrichment Analysis (GSEA) of the TCGA UVM cohort (n = 80) using Hallmark and Reactome gene sets from MSigDB reveals clinical relevance of key pathways identified in resistance mechanisms under FAK or MEK inhibition. (**A**) High activity of the PI3K/AKT/mTOR pathway is associated with significantly worse overall survival (*p* = 0.0051). (**B**) Elevated mTORC1 signaling correlates with poor survival outcomes (*p* = 0.0021). (**C**) High anti-apoptosis pathway activity is linked to reduced survival (*p* = 0.0076). (**D)** Increased IL6/JAK/STAT signaling is associated with worse survival (*p* < 0.0001). (**E**) High YAP signature activity predicts poorer survival outcomes (*p* = 0.0019).

Our GSEA revealed that high expression of the Hallmark PI3K/AKT/mTOR pathway (Figure 4A) or Hallmark mTORC1 signaling (Figure 4B) correlated with significantly worse overall survival (*p* = 0.0051 and *p* = 0.0021, respectively), aligned with our in vitro data that mTOR pathway activation drives therapeutic resistance. Moreover, tumors with a high anti-apoptosis gene signature (Figure 4C) showed markedly reduced survival (*p* = 0.0076), in line with our identification of BCL2/BCL-XL upregulation as a key resistance mechanism. High IL6/JAK/STAT signaling (Figure 4D) was even more strongly associated with adverse outcomes (*p* < 0.0001), further underscoring that JAK/STAT blockade may represent an important complementary strategy in UVM therapy. Notably, a high YAP signature (e.g., CTGF, CYR61, and other canonical YAP targets) (Figure 4E) also tracked with significantly inferior survival (*p* = 0.0019), highlighting the clinical importance of Hippo pathway dysregulation in UVM.

Collectively, these results underscore that the same pathways identified in our mechanistic screen, PI3K/AKT/mTOR, anti-apoptosis (BCL2 family), IL6/JAK/STAT and Hippo/YAP, are not only active in primary UVM tumors but are also associated with worse survival profiles in large patient cohorts (n = 80).

### 3.4. Correlating Key Resistance-Associated Genes with Patient Survival in Uveal Melanoma

**Figure 5:**
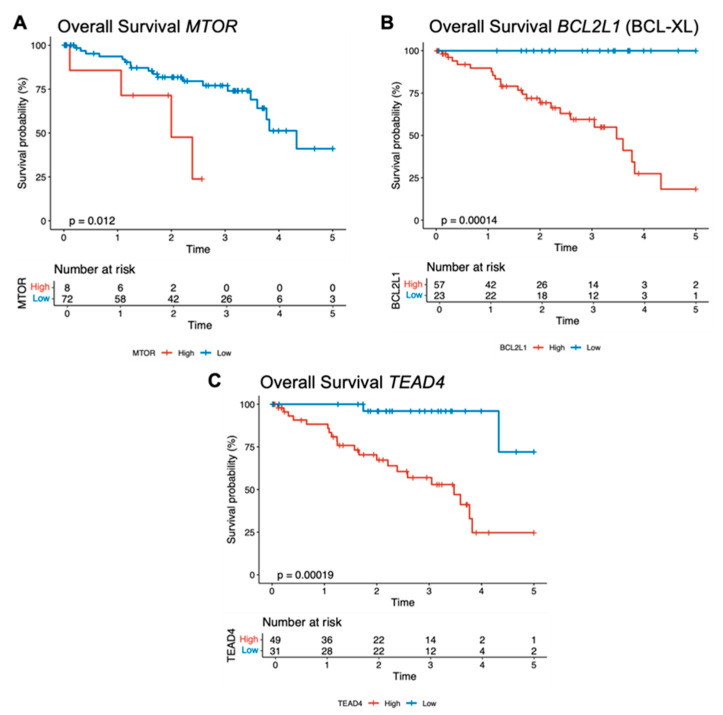
Elevated expression of mTOR, BCL2L1 (BCL-XL), and TEAD4 correlates with worse overall survival. Kaplan–Meier survival curves illustrate the difference in patient outcomes in the TCGA Uveal Melanoma cohort (n = 80) when classified into high (red) versus low (blue) transcript expression groups for each gene. (**A**) High mTOR expression is associated with reduced overall survival (log-rank *p* = 0.012). (**B**) High BCL2L1 expression (anti-apoptotic BCL-XL) significantly predicts poorer survival (log-rank *p* = 0.00014). (**C**) High TEAD4 expression, reflecting active Hippo–YAP/TEAD signaling, correlates with inferior patient outcomes (log-rank *p* = 0.00019). Numbers at risk for each subgroup are shown below the *x*-axis.

To investigate the translational implications of these observations, we next assessed whether expression levels of key resistance-associated genes correlated with patient outcomes in a cohort of 80 individuals diagnosed with UVM. Focusing on MTOR, BCL2, and TEAD, each patient was categorized as having “high” or “low” transcript levels (TPM). The Kaplan–Meier survival curves uncovered distinct patterns among the three genes (Figure 5). High MTOR expression was associated with a steep decline in overall survival (OS) between 20 and 60 months (log-rank *p* = 0.044). By five years, survival in the high-MTOR group approached 20%, whereas the low-MTOR cohort exceeded 60%. These data highlight a potential role for upregulated mTOR signaling in aggressive disease phenotypes and underscore the plausibility of integrating mTOR inhibitors with MEKi or FAKi to counteract emergent resistance (Figure 5A).

Analysis of BCL2L1, a key anti-apoptotic gene, showed elevated transcript levels were linked with significantly worse survival (*p* = 0.04), suggesting that tumors with heightened anti-apoptotic capacity may rapidly evade cancer therapies (Figure 5B). Targeting BCL-XL (e.g., via BH3 mimetics) combined with existing inhibitors could disrupt this powerful survival shield. Notably, high TEAD4 expression was associated with a pronounced risk of mortality (log-rank *p* = 0.00087), with a hazard ratio of 4.8 (*p* = 0.0025), highlighting the critical involvement of Hippo pathway effectors in driving UVM aggressiveness and potential therapy escape (Figure 5C).

Although UVM is predominantly driven by GNAQ/GNA11 mutations, these data illuminate an additional, non-mutational layer of plasticity that may arise during therapy. Taken together, our screen and correlative analyses delineate potential biomarkers for patient selection or exclusion (based on high expression of MTOR or BCL2L1), real-time monitoring of therapeutic response (assessing upregulation of PI3K/mTOR or BCL2-family members), and design of next-generation combination strategies aimed at delaying or preventing resistance. Indeed, prior work in other malignancies supports that concurrent PI3K/mTOR inhibition can enhance the efficacy of MEK blockade [29,30]. Our findings now extend these insights to UVM and underscore the translational relevance of targeting these pathways (e.g., with PI3K/mTOR inhibitors, BH3 mimetics, or JAK inhibitors) alongside MEK or FAK blockade to prevent or delay resistance.

### 3.5. Aberrant Activation of the AKT and YAP Pathways Induces Resistance to the Antiproliferative Effects of MEKi and FAKi

**Figure 6:**
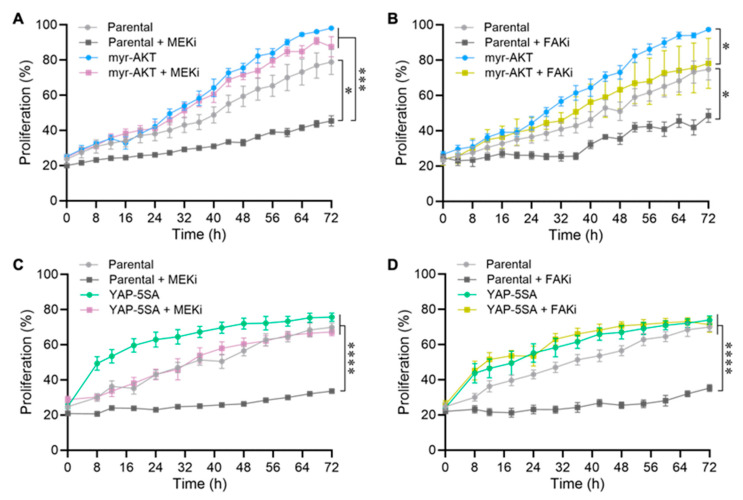
Expression of myr-AKT and YAP-5SA oncoproteins promotes resistance to MEKi and FAKi. (**A**) Proliferation assays of parental cells and myr-AKT with or without treatment with MEKi (3 nM, trametinib) or (**B**) with FAKi (0.3 μM, VS-4718). Two-way repeated measures ANOVA followed by Tukey post hoc, * *p* < 0.05, *** *p* < 0.001. (**C**) proliferation of parental and YAP-5SA cells treated with MEKi (3 nM, trametinib) or (**D**) with FAKi (0.3 μM, VS-4718). Two-way repeated measures ANOVA followed by Tukey post hoc, **** *p* < 0.001.

**Figure 7:**
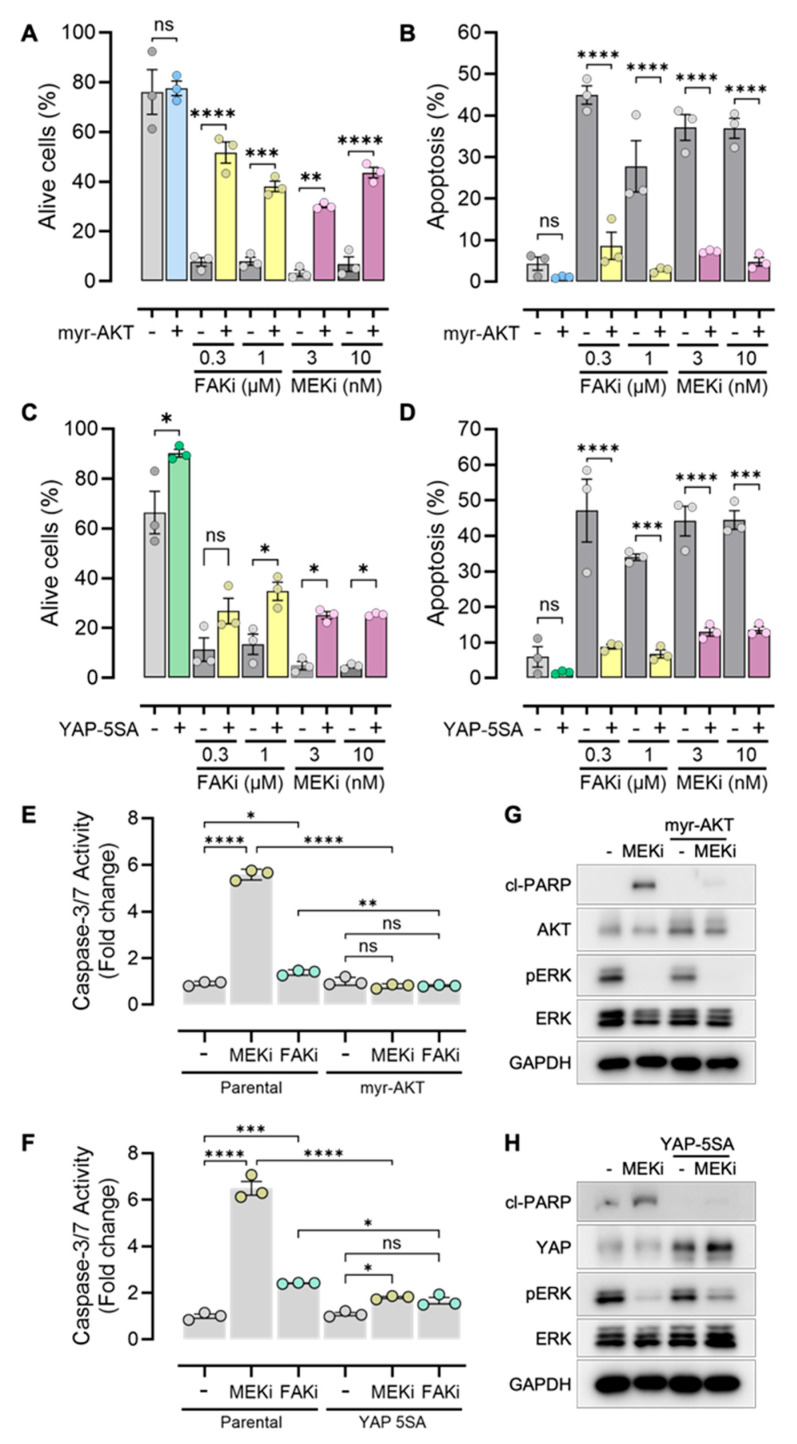
Activation of YAP and AKT pathways promotes resistance to apoptosis induced by MEK and FAK inhibitors. (**A**) Quantification of live cells and (**B**) apoptotic cells treated with FAKi or MEKi in parental cells and myr-AKT cells. FAKi (VS-4718) 0.3 and 1 μM; MEKi (trametinib) 3 and 10 nM. One-way ANOVA followed by Tukey post hoc; ns, not significant, ** *p* < 0.01, *** *p* < 0.001, **** *p* < 0.0001. (**C**). Quantification of live cells, (**D**) apoptotic cells treated with FAKi or MEKi in parental cells and YAP-5SA cells. FAKi (VS-4718) 0.3 and 1 μM; MEKi (trametinib) 3 and 10 nM. One-way ANOVA followed by Tukey post hoc; ns, not significant, * *p* < 0.05, *** *p* < 0.001, **** *p* < 0.0001. (**E**) Caspase-3/7 activity assays in parental, myr-AKT, and (**F**) YAP-5SA cells treated with MEKi (10 nM, trametinib) and FAKi (1 μM, VS-4718). One-way ANOVA followed by Tukey post hoc; ns, not significant, * *p* < 0.05, ** *p* < 0.01, *** *p* < 0.001, **** *p* < 0.001. (**G**) Western blot of cl-PARP in myr-AKT and (**H**) YAP-5SA expressing cells treated with MEKi (10 nM, trametinib) by 24 h. The uncropped blots are shown in Figure S3.

Building on our results, we selected the two most representative candidate oncogenic mechanisms to explore the effect of aberrant PI3K/AKT (myr-AKT) and YAP/TEAD (YAP-5SA) pathway activation on resistance to the cytotoxic effects of FAKi and MEKi. We initially analyzed the proliferation of parental 92.1 cells and myr-AKT and YAP-5SA-expressing cells treated with FAKi or MEKi (Figure 6A–D) (see Western blots below, Figure 7). We observed that myr-AKT and YAP-5SA-expressing cells resist FAKi and MEKi treatment, proliferating efficiently in contrast to parental cells, where proliferation was inhibited by these treatments. Interestingly, the proliferation of myr-AKT cells was significantly greater than that of parental cells (Figure 6B). Aligned with these findings, cell migration in wound-closure assays showed that the oncoproteins myr-AKT and YAP-5SA also increase cell motility in serum-containing medium (Supporting Figure S1). At the same time, FAKi decreased cell migration and MEKi increased cell motility, consistent with the previously reported increased FAK activation in response to MEK inhibitors in uveal [25] and cutaneous melanoma [31]. The AKT and YAP pathways did not affect the effect of FAKi and MEKi on cell migration, in contrast to promoting resistance to the anti-proliferative effects of these treatments (Supporting Figure S1).

### 3.6. Myr-AKT and YAP-5SA Protect UVM Cells from FAKi- or MEKi-Induced Apoptosis

We hypothesized that the protective effects of myr-AKT and YAP-5SA in UVM cells against the cytotoxicity of FAKi and MEKi may result from counteracting their effectiveness in promoting cell death. We evaluated the impact of FAKi and MEKi on apoptotic cell death using ethidium bromide and acridine orange fluorescence staining of cells, where the live cells stain only the nucleus with green fluorescence (acridine orange), in contrast the apoptotic cells have green and red fluorescence (acridine + ethidium bromide) because the integrity of the plasma and nuclear membrane was compromised (Supporting Figure S2). FAKi and MEKi drastically decreased the percentage of parental 92.1 live cells, with the majority of cells showing membrane permeability for ethidium bromide influx (Figure 7A,C, Supporting Figure S2). When cells express myr-AKT or YAP-5SA and are treated with FAKi and MEKi, the apoptotic cells decreased significantly (Figure 7B,D). Subsequently, we also analyzed caspase 3/7 activity. MEKi increased caspase activity in the treated parental cells, while FAKi, as a single agent, was less effective. UVM cells expressing myr-AKT (Figure 7E) and YAP-5SA (Figure 7F) were resistant to the induction of caspase by MEKi, suggesting that the oncoproteins promote pro-survival pathways resulting in reduced caspase activation. Consistently, when analyzing cleaved-PARP, we confirmed that myr-AKT (Figure 7G) and YAP-5SA (Figure 7H) reduce cell death, without considerably altering the effect of MEKi on reducing pERK; therefore, the AKT and YAP pathways induce resistance mechanisms independent of ERK reactivation.

## 4. Discussion

Targeted therapies are essential in precision oncology, but their success in uveal melanoma is limited by the rapid emergence of adaptive resistance. By screening 100 active or dominant-negative mutants resulting in persistent activation of cancer-associated signaling nodes in a BAP1 wild-type UVM model (92.1) and validating the top hits in a BAP1-mutated cellular model (MP46), we characterized potential signaling mechanisms that modulate sensitivity to GNAQ-pathway inhibition. While the use of ectopic gene expression in UVM cells may have some limitations with respect to the activation of endogenous signaling events; nonetheless, this approach, this approach allowed for the identification of pathways whose activation is sufficient to attenuate drug response under these defined experimental conditions.

Earlier clinical trials with MEK inhibitors have shown limited benefit [19,20,21]. Accumulating evidence suggests that adaptive compensatory signaling may contribute to the insufficient therapeutic effect. Our study highlights several canonical and non-canonical signaling pathways that, when experimentally activated, are capable of modulating sensitivity to MEK and FAK inhibition, outlining a network of potential adaptive signaling responses in uveal melanoma cells.

Among the pathways identified, the Hippo/YAP pathway appeared as a notable modulator of drug sensitivity. The oncogenic role of YAP in UVM is well established [8,32]. In the context of our in vitro models, we observed that YAP hyperactivation, either through loss of upstream Hippo pathway regulator LATS or directly activated by expression of a phosphorylation-defective YAP mutant, was associated with reduced sensitivity to FAK and MEK inhibition. These findings are consistent with YAP/TEAD-mediated transcription acting as a downstream transcriptional output for multiple signaling inputs associated with reduced drug sensitivity in our experimental models.

Our results also highlight the PI3K/AKT/mTOR axis in the modulation of response to both MEK and FAK inhibition. Previous studies suggested a role for AKT/mTOR reactivation in limiting the activity of MEK inhibitors [33], but which component within this pathway could restore survival signaling functionally remained unclear. Through an unbiased screen, we observed that constitutive activation of this pathway (e.g., by myr-AKT and mTOR variants) is sufficient to partially restore cell viability under combined inhibition in both BAP1-wildtype and BAP1-mutant cell lines. These findings provide a mechanistic rationale for further investigating combination strategies that concurrently target MEK, FAK, and mTOR and may help clarify the synergy reported in previous in vivo studies [33].

In addition to signaling pathways regulating proliferation and survival, our data indicate that modulation of apoptotic thresholds can influence drug response. Specifically, overexpression of BCL2 or BCL-XL protected UVM cells from therapy-induced cell death in our assays. This is consistent with the sensitivity of UVM patient-derived xenograft (PDX) models to BH3 mimetics in [34]. In addition, our data demonstrate that BCL-2 family upregulation is not only associated with resistance but also a functional determinant of cancer cell survival in vitro that can be pharmacologically reversed. In this regard, we have recently demonstrated the increase in BCL-XL, BCL2, and MCL1 during the treatment with MEK and FAK inhibitors [35]. Specifically, we observed that BCL2 promotes resistance to MEKi and FAKi combination, which can be exploited pharmacologically using BCL2 inhibitors (venetoclax) that act synergistically to promote UVM cell death [35]. Therefore, BCL-2 inhibition represents a promising strategy to convert the cytostatic effect of current therapies into a cytotoxic response. In addition, emerging data suggest that BCL-XL expression may contribute to resistance to FAKi and MEKi, in conjunction with BCL2.

Limitations and future directions: In summary, this study examines the signaling plasticity of uveal melanoma cells by systematically mapping pathways that allow cells to evade growth suppression and cell death upon MEK and FAK inhibition in controlled in vitro experimental settings. We report multiple resistance mechanisms (including activation of PI3K/mTOR, BCL-2 family proteins, YAP/TEAD, and potentially JAK/STAT) within a coherent framework, and delineate specific signaling nodes that modulate cellular responses to pathway inhibition.

The concordance between our findings and the adaptive mechanisms reported across multiple UVM models supports the biological relevance of the pathways identified in our screen, yet we acknowledge that the present analyses were performed in a limited number of cell lines and relied on ectopic expression levels that may exceed physiological thresholds. In addition, our experimental approach relied exclusively on in vitro models and did not include in vivo or patient-derived systems, which will be important to further evaluate the relevance of these findings in more complex biological contexts

Going forward, it will be crucial to validate our findings across additional UVM models and expanded patient cohorts to prioritize the most tractable molecular targets and develop multimodal therapeutic strategies that aim to prevent resistance before it emerges.

## 5. Conclusions

These insights pave the way for the development of customized therapeutic regimens that anticipate and minimize resistance, creating an opportunity for deeper and more durable responses and potentially curative outcomes in patients with advanced UVM. Notably, by focusing on validating the most significant results in MP46 (BAP1-mutated) UVM cells, we observed consistent mechanisms of resistance, reinforcing the notion that targeting shared compensatory pathways (e.g., BCL-2 family, PI3K/mTOR, YAP/TEAD) alongside *GNAQ*/*GNA11*-driven signaling networks represent a critical strategy for advancing UVM therapy.

## Figures and Tables

**Table 1:** List of genes included in the mutant library screen, grouped by pathways involved in resistance mechanisms, including PI3K/AKT/mTOR, RAS/MAPK, TGF-β/SMAD, NF-κB, and apoptosis pathways.

Pathway	Genes and Expression Constructs
PI3K/AKT/mTOR Pathway	*AKT1*, *MTOR*, *PTEN*, *PIK3CA*, *PIK3CG*, *PIK3R1*, myr-FLAG-PI3K, myr-FLAG-Akt, FLAG-Rheb (Q64L)
RAS/MAPK Pathway	*BRAF*, *KRAS*, *NRAS*, *MAP2K1*, *MEK1*, *MAP2K4*, *MEK1* (S218D/S222D), HRas (G12V), Kras (G12V), JNK2 WT O/E (MAPK9), p38 WT O/E (MAPK14), MKK6(S207E/T211E), Mkk7-JNK2 fusion
Cell Cycle Regulation	*CCND1*, *CDKN1A*, *CDKN1B*, *RB1*, *STAG2*
Receptor Tyrosine Kinases	*EGFR*, *FGFR2*, *FGFR3*, *FLT3*, *PDGFRA*, *RET*, *HGF*
WNT/β-catenin Pathway	*CTNNB1*, β-catenin (S33A, S37A, T41A), β-catenin (S33Y), GSK3b(K85A)
Notch Signaling	Notch1 intracell.domain, Notch3 intracell.domain
TGF-β/SMAD Pathway	*SMAD2*, *SMAD4*, *ACVR1B*, *TGFBR1* (T204D)
Epigenetic Regulation	*EZH2*, *HIST1H1C*, *H3F3B*, *KLF4*, *BAP1*, *TET2*
DNA Damage Response	*CHEK2*, *MLH1*, *MSH2*
Oxidative Stress Response	*NFE2L2*, *KEAP1*
Apoptosis	*BCL2*, *BCL-XL*, Caspase-8 (C360A), Caspase-3 (C163A)
Transcription Factors	*FOXA1*, *ELF3*, *GATA2*, *GATA3*, *RUNX1*, *VHL*
NF-κB Pathway	IKKa (S176E/S180E), FLAG-IKKb (S177E/S181E), *CYLD*, *MYD88*
RNA Splicing Pathway	*U2AF1*
Jak/Stat Pathway	JAK2 (V617F), C-term-FLAG-Stat3(A661C/N663C)
JNK Pathway	JNK2 WT O/E (*MAPK9*), Mkk7-JNK2 fusion
ERK5 Pathway	MEK5 DD, myr-MEK5
p38 MAPK Pathway	p38 WT O/E (*MAPK14*), MKK6(S207E/T211E)
Hedgehog Pathway	Gli2 truncation, 4x myc Gli2 truncation, SmoM2
Estrogen Receptor Pathway	ERα (Y537S mutant)
Hippo Pathway	YAP2 5SA, LATS2(K697R) kinase dead
P53 Pathway	p53 (R175H mutant DN)
Ral-GEF Pathway	H-Ras (G12V, E37G), Rgl2-CAAX, RalA(G23V)(two forms—full and mature peptide), *MEK1*

## Data Availability

The data generated and analyzed during the current study are available from the corresponding author upon reasonable request.

## Supplementary Materials

The following supporting information can be downloaded at https://www.mdpi.com/article/10.3390/cancers18010074/s1, Table S1: MEKi screen shows Cell Viability Max Response of individually virally transduced cell populations with mutant library in 92.1 cells; Table S2: FAKi screen shows Cell Viability Max Response of individually virally transduced cell populations with mutant library in 92.1 cells. Figure S1: The myr-AKT and YAP5SA oncoproteins increase cell migration in wound closure assay; Figure S2: Cytotoxicity of FAKi and MEKi in myr-AKT and YAP-5SA 92.1 cells; Figure S3: Uncropped Western blot images corresponding to the blots shown in Figure 7.

## Abbreviations

The following abbreviations are used in this manuscript:

UVM	Uveal melanoma
FAK	Focal adhesion kinase
GSEA	Gene Set Enrichment Analysis
MSigDB	Molecular Signatures Database
