# Supplementary material for: Unraveling Resistance Mechanisms to Gαq Pathway Inhibition in Uveal Melanoma: Insights from Signaling-Activation Library Screening
Source: Cancers (Basel). 2025 Dec 25;18(1):74. doi: 10.3390/cancers18010074 (PMC12785119; doi:10.3390/cancers18010074)

Supporting Figure S1. The myr-AKT and YAP5SA oncoproteins increase cell migration in wound closure assay.

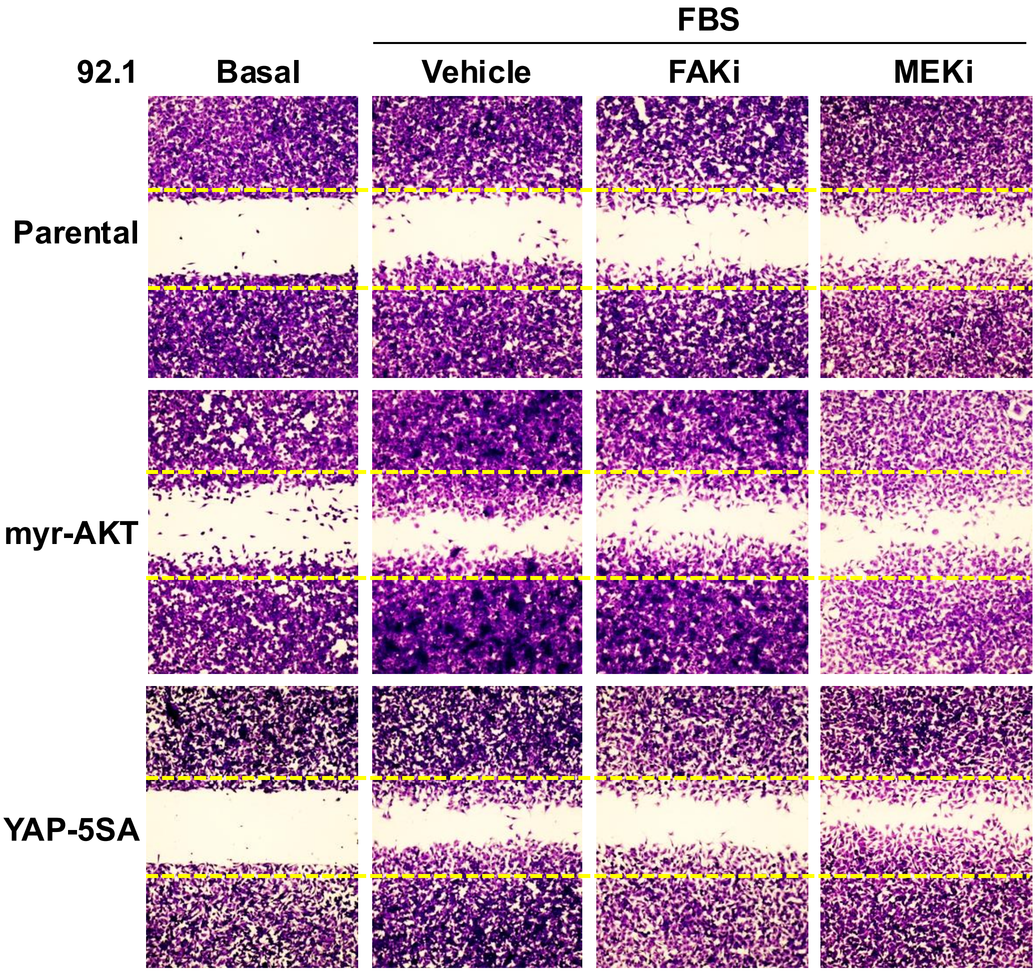

Supporting Figure S1. The myr-AKT and YAP5SA oncoproteins improve cell migration in the wound closure assay. Cell migrations in wound closure were performed with 92.1 parental, myr-AKT and YAP-5SA cells on gelatin 0.02%. Cells were treated with FAKi (1 μM, VS-4718) and MEKi (1 μM, trametinib).

Supporting Figure S2. Cytotoxicity of FAKi and MEKi in myr-AKT and YAP-5SA 92.1 cells

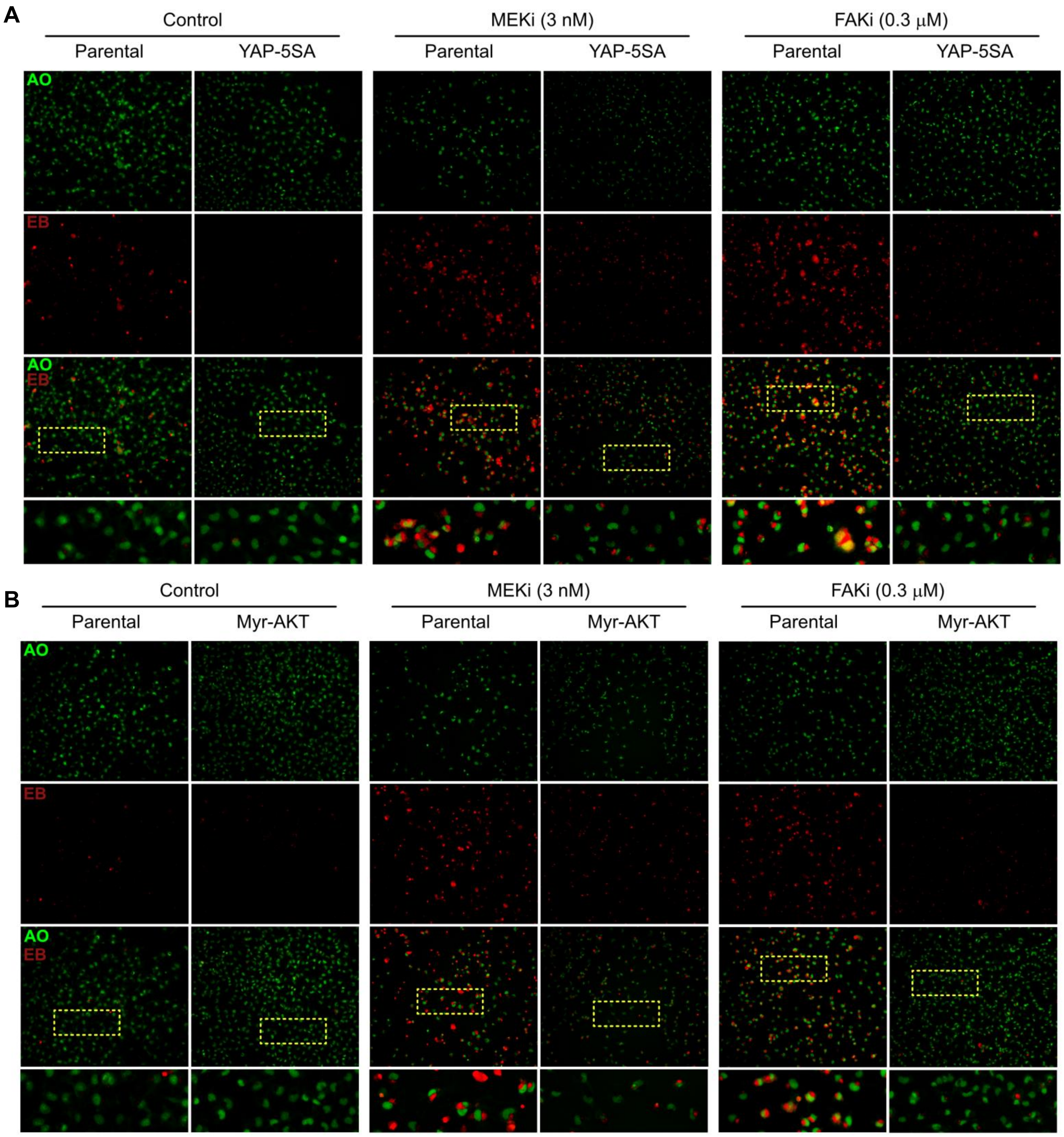

Supporting Figure S2. Cytotoxicity of FAKi and MEKi in myr-AKT and YAP5SA expressing 92.1 cells. A) Fluorescence of 92.1 cells stained with acridine orange (AO) and ethidium bromide (EB). Representative fluorescence of 92.1 parental, YAP5SA, and B) myr-AKT expressing cells treated with FAKi (1 μM, VS-4718) and MEKi (1 μM, trametinib). Related to Figure 7A-7F.

Supporting Figure S3. Uncropped Western blot images corresponding to the blots shown in Figure 7

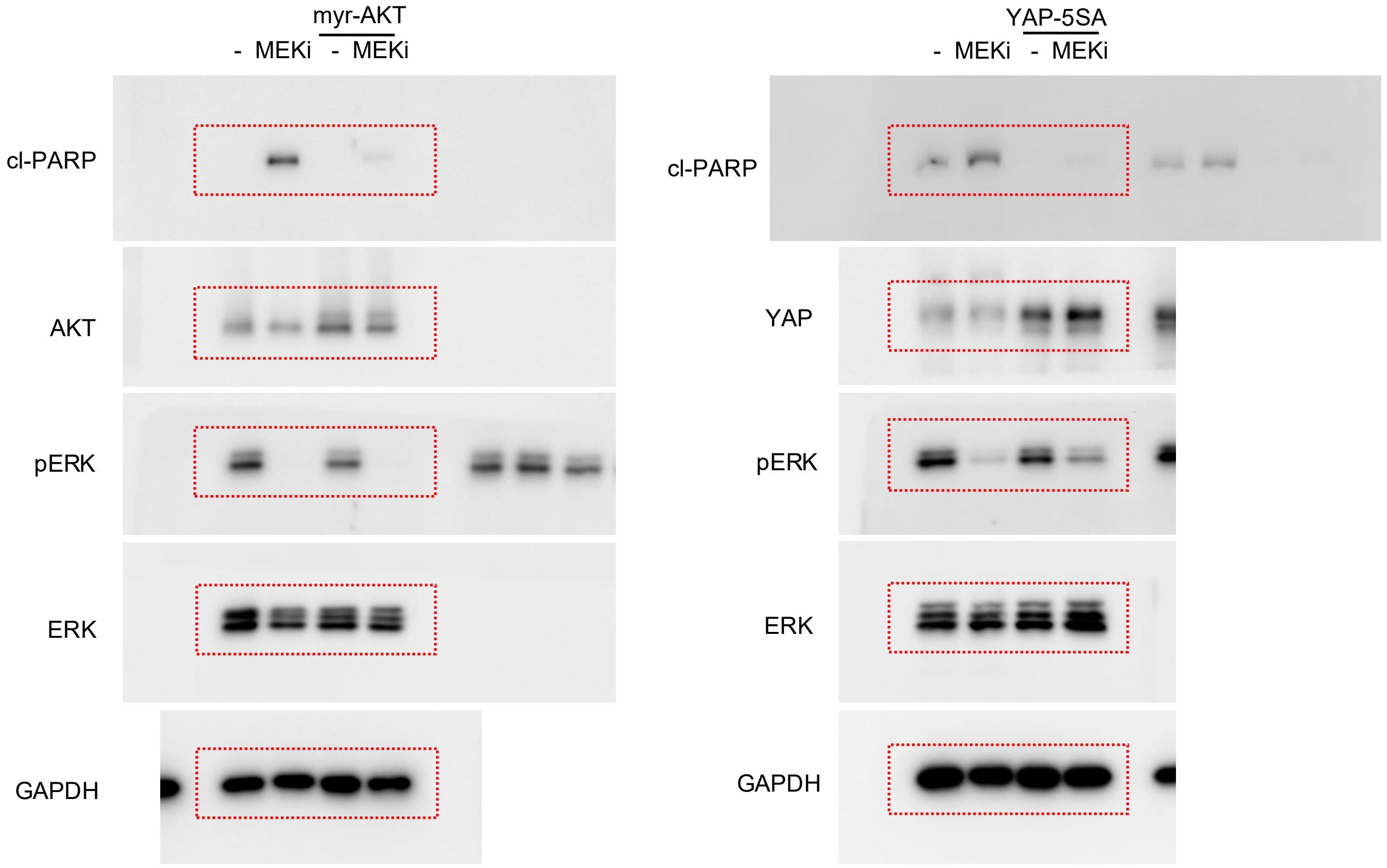

Supplement: Supplementary file 1 [file cancers-18-00074-s001.zip › Supplementary Figures.pdf]
